# Supplementary material for: Rapid detection of microbiota cell type diversity using machine-learned classification of flow cytometry data
Source: Commun Biol. 2020 Jul 15;3:379. doi: 10.1038/s42003-020-1106-y (PMC7363847; doi:10.1038/s42003-020-1106-y)
Supplement: Supplementary file 1 — Supplementary Information [file 42003_2020_1106_MOESM1_ESM.pdf]

Supplementary Information

Table of content **page**

Supplementary Figures

Supplementary Figure 1 3
Supplementary Figure 2 4
Supplementary Figure 3 5
Supplementary Figure 4 6
Supplementary Figure 5 7
Supplementary Figure 6 8
Supplementary Figure 7 9
Supplementary Tables
Supplementary Table 1 11
Supplementary Table 2 13
Supplementary Notes
Glossary of Terms 14
Supplementary Methods
Section 1. Data pretreatment 16
1.1 Filtering of FCM data 16
1.2 Gating 17
Section 2. Artificial neural network reconstruction. 18
2.1. Subsampling and anchoring 19
2.2. ANN selection, training and validation 19
2.3 Analysis of Fig. 2c, confusion plot 19
Section 3. CellCognize testing of standard-mixed communities. 20
3.1 Preparing a limited ANN with five standards only. 20
3.2 Analyze the synthetic community mixtures. 20
3.3. Analysis of Figure 2a. 21
3.4 PCA analysis of Figure 2b. 22
3.5 Analysis of Figure 2d. 23
3.6 Run ANN-32 classifications 23
3.7 In-silico mixture of four E. coli strains with lake water background. 24
3.8 Analysis of Figure 2e: Class attribution of aquatic microbial community. 25
3.9 In silico mixing three standards into lake water: Fig. 2e lower panel. 26
3.10 Fig. 2f Analysis. 27
Section 4. Lake water microbial community enrichment 28
4.1 Analysis Fig. 3a and b 28
4.2 Analysis of Fig. 3c 29
4.3 Analysis of Fig. 3d 29
Section 5. Analysis of similarity scores using CellCognize. 29
5.1 Calculate mean probabilities of standards in their class attribution (Fig. 4a) 29
5.2 Calculate mean probability per assigned class in lakewater. 30
5.3 Analysis of Fig. 4b. Probability distributions 31
5.4 Analysis of Fig. 4c. Mean class attribution and similarity scores in the 1-octanol enriched lake water community, using the ANN-32 classifiers. 32
5.5 Mean class attribution and similarity scores using an ANN-33 classifier 34

Supplementary Reference 35

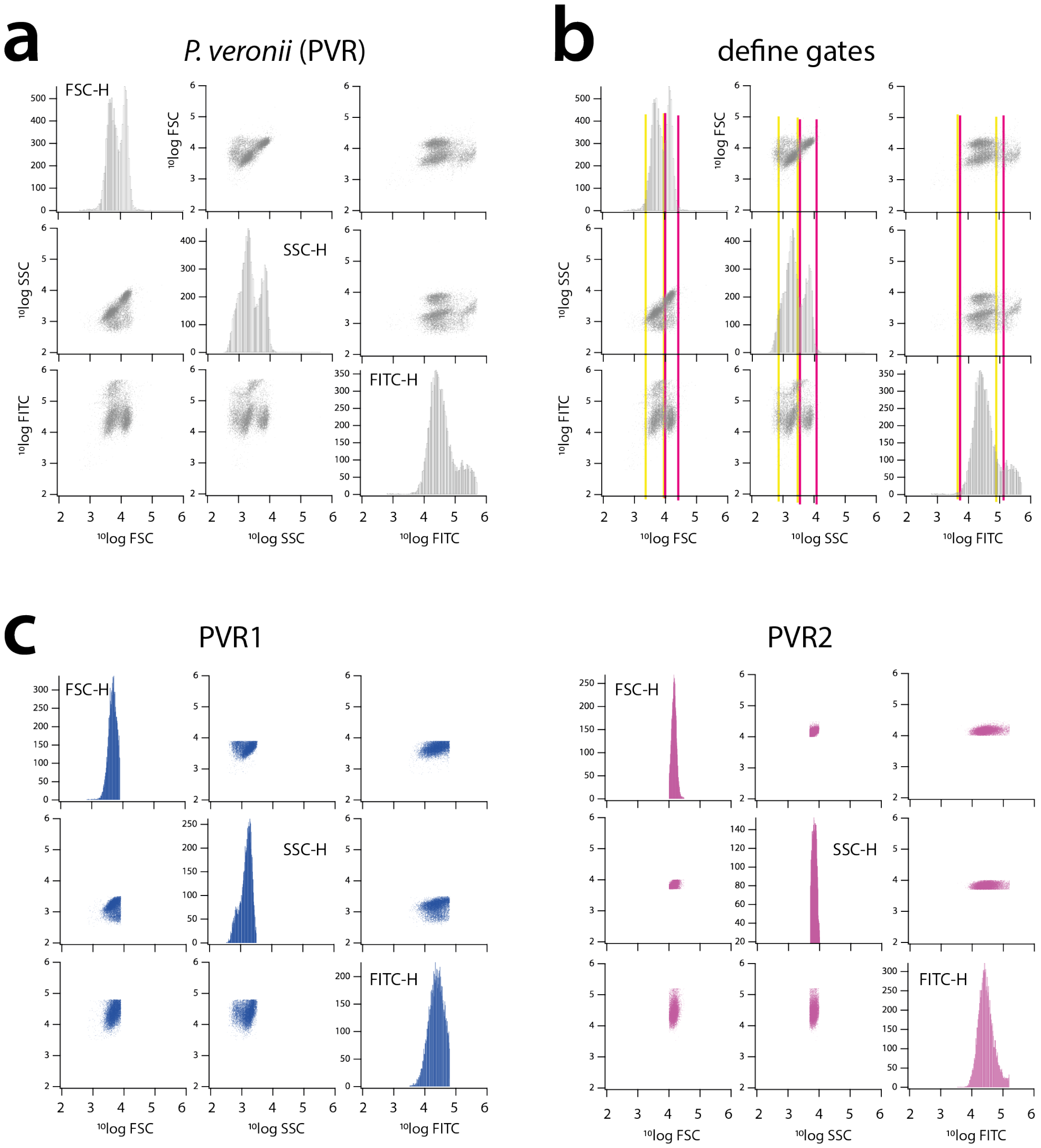

Supplementary figure 1. Illustration of the strain standard gating process. a Raw FCM data of the FSC-height, SSC-height and FITC-height channels for the standard PVR (*Pseudomonas veronii* stained with SybrGreen I), subsampled to 20,000 events for ease of plotting. Note the two visible subpopulations in the histograms of each channel. **b** Subpopulations are gated (separated) by imposing respective minima and maxima values on each of the log-scales. **c** The resulting two subpopulations PVR1 and PVR2 were included as separate standards in the training, validation and testing of the ANN classifier. Note that the fluorescence in the FITC-H channel originates from SYBR Green I staining.

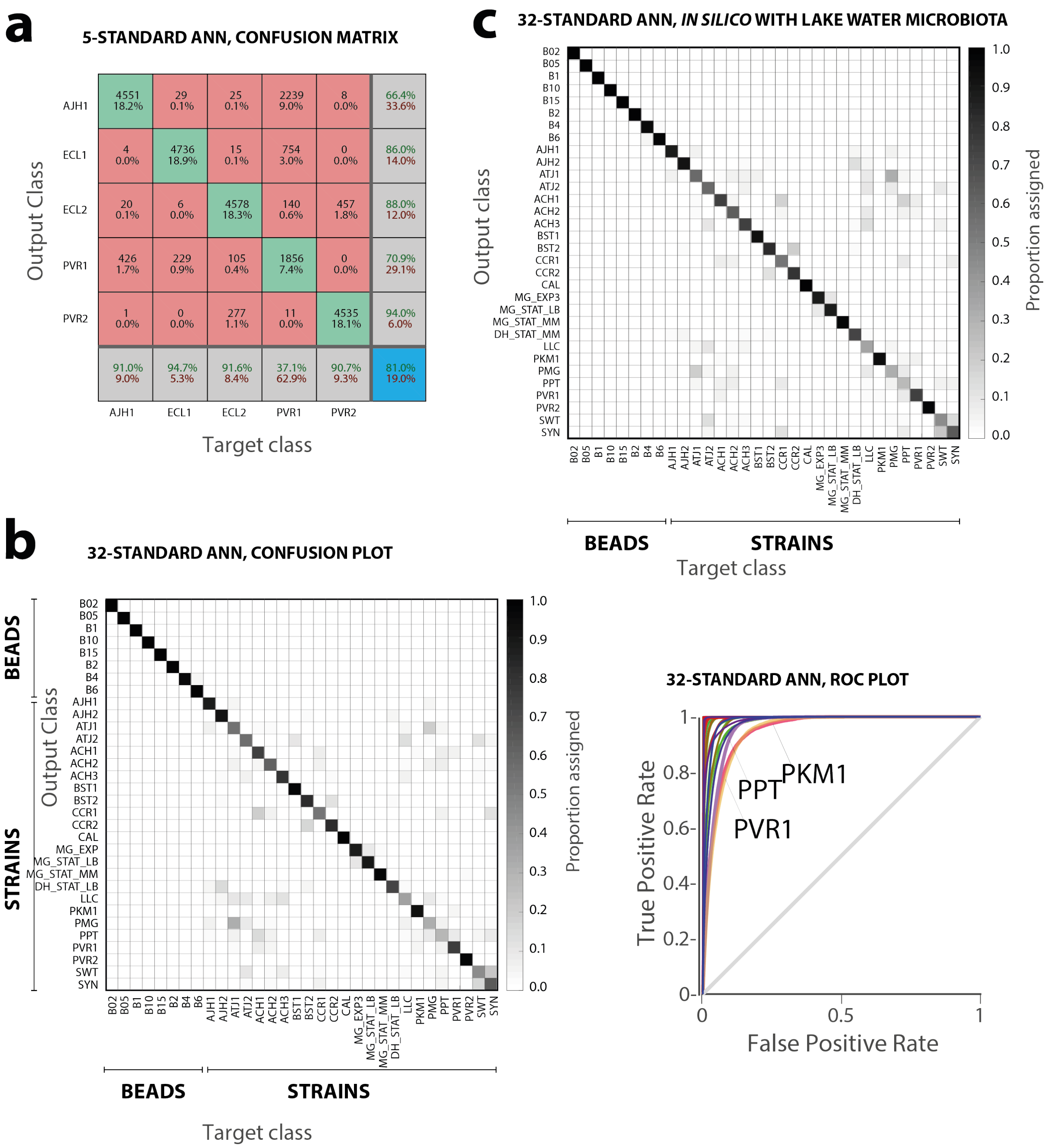

Supplementary figure 2. Confusion plots of five- and 32-standard ANNs. a ANN classifier with five classes covering the five subpopulations from the three strains, *A. johnsonii* (AJH), *E. coli* MG1655 (ECL1 and ECL2), and *P. veronii* (PVR1 and PVR2). The confusion matrix shows absolute numbers of events assigned to each of the classes for a dataset consisting of *n* = 5000 randomly subsampled FCM data for each of the standards. "The rows correspond to the predicted class (Output Class) and the columns correspond to the true class (Target Class). The diagonal cells correspond to observations that are correctly classified. The off-diagonal cells correspond to incorrectly classified observations. Both the number of observations and the percentage of the total number of observations are shown in each cell. The column on the far right of the plot shows the percentages of all the examples predicted to belong to each class that are correctly and incorrectly classified. These metrics are often called the **precision** (or positive predictive value) and false discovery rate, respectively. The row at the bottom of the plot shows the percentages of all the examples belonging

to each class that are correctly and incorrectly classified. These metrics are often called the **recall** (or true positive rate) and false negative rate, respectively. The cell in the bottom right of the plot shows the overall **accuracy**." [1] **b** Confusion matrix and ROC plot for one of the 32-standard ANN classifiers showing mean prediction accuracy of the classifier (as grey-level, according to the scale bar on the right) for the precision (rows) versus the recall (columns) for a dataset consisting of *n* = 5000 randomly subsampled and merged FCM data for each of the 32 standards. Abbreviations for the standards are given in Table 1. Numerical averages across all five independent ANN runs are reported in Table 1. Receiver Operating Characteristics (ROC) curves show the predicted false positive rate at expected true positive rate for each strain or bead standard (differently colored lines), with three labeled curves for standards that have the highest false positive rates at 80% true positives. Note that ROC curves toward the upper left corner indicate higher probability for true positive classification at low expected false positive rate. The grey diagonal line represents random classification for any single class. **c** HeatMap matrix showing recovery rates from the five 32-standard ANN classifiers for each of the standards with n=5000 cells *in silico* mixed within a background of the freshwater microbial community with n=5038 cells. Recovery rates were calculated as the mean percentage of each standard attributed to its own class after subtracting the background lake water cells: (Absolute cell counts of class attribution – absolute cell counts of freshwater assigned to that class)/added cells (n=5000).

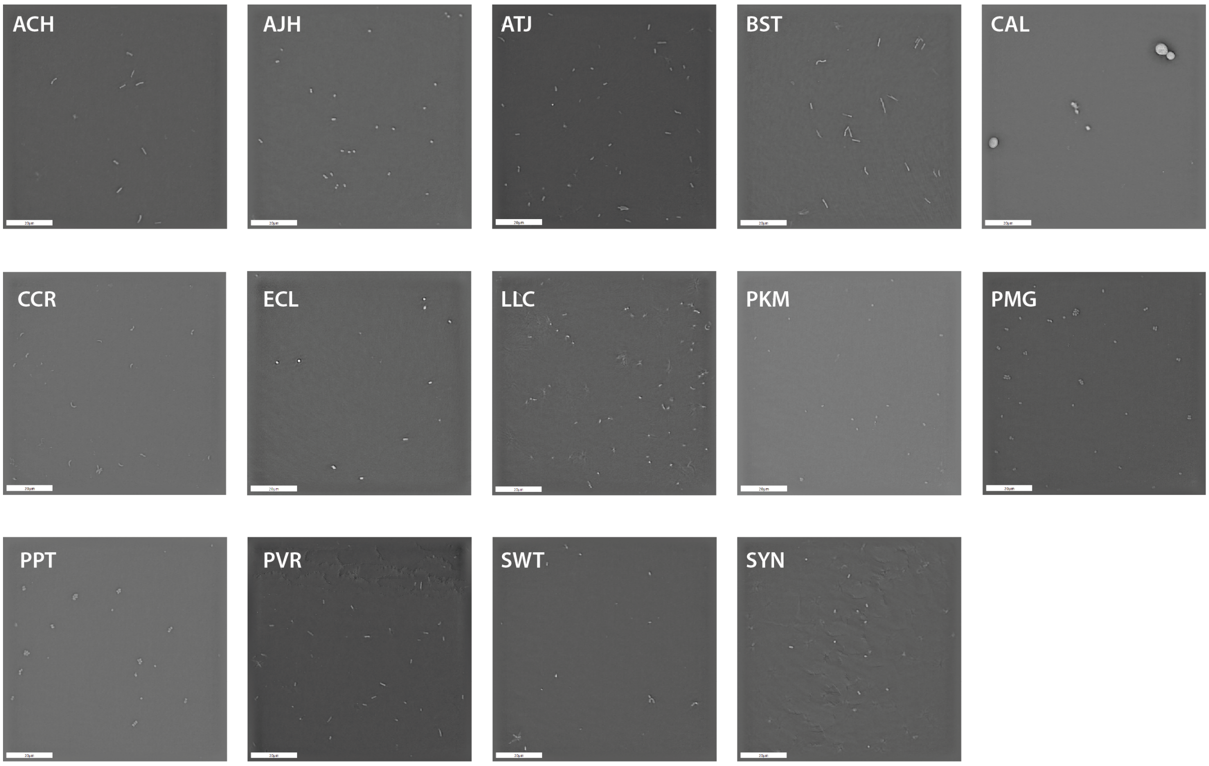

Supplementary figure 3. Planar representation of a 1 µm-stack of holographic imaging of strain standards used in the FCM-ANN pipeline. Scale bars are 20 µm. “White” cell outlines result from the recognized cell shape boundaries in the holographic imaging software (*Materials and methods*). Calculated mean cell volumes reported in Supplementary Table 2.

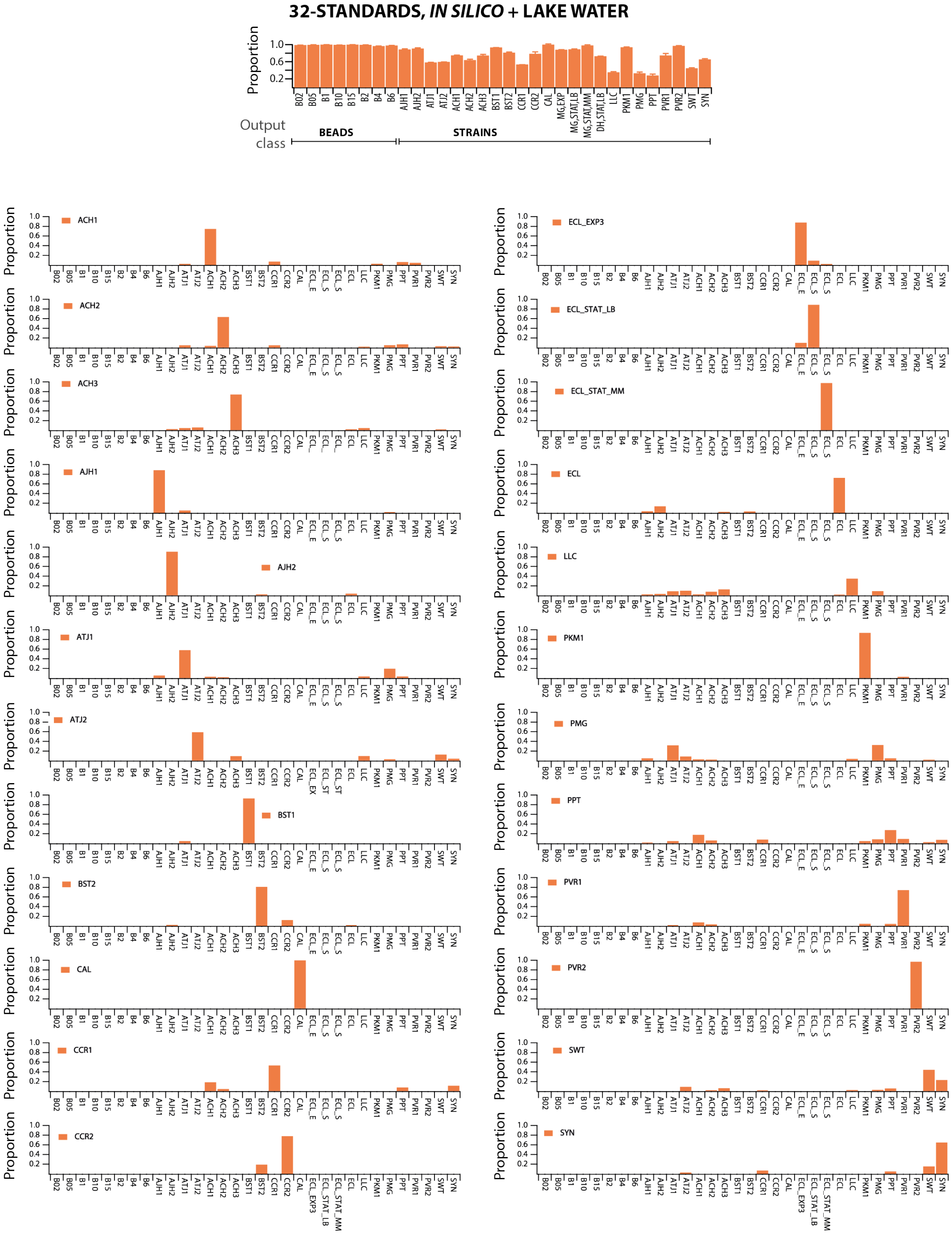

Supplementary figure 4. Correct predicted classification of cell standard data added *in silico* to a Lake Geneva microbiota background. Panels show the collective (top) and individual predicted classification (as proportion) of 5000 randomly subsampled FCM data from the strain standards, combined *in silico* with 5036 events from FCM analysis of the lake water microbiota.

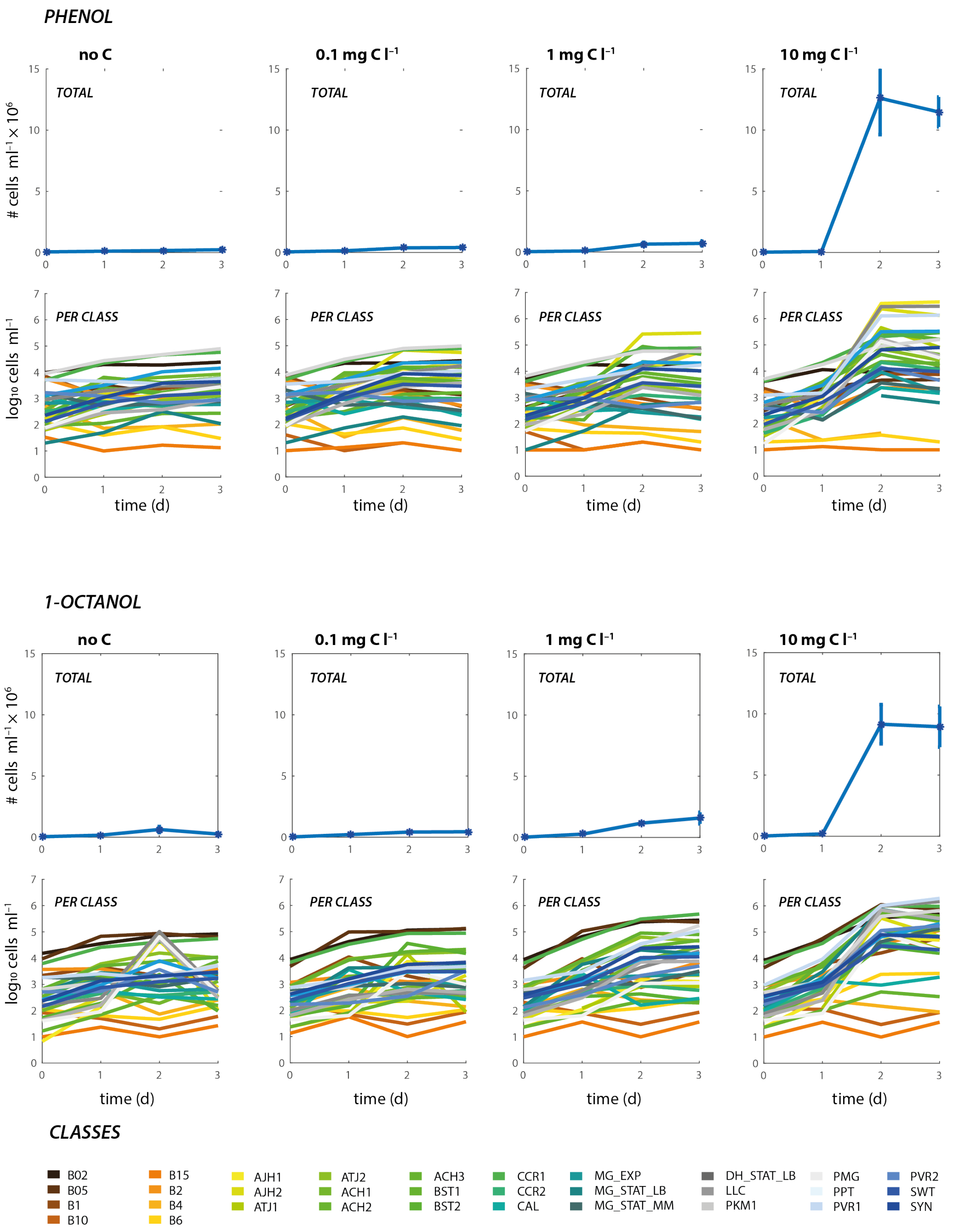

Supplementary figure 5. CellCognize subpopulation growth of Lake Geneva freshwater communities upon substrate amendment. Panels show total community size (as $\log_{10}$ cells ml^{-1}) and the abundance of CellCognize-attributed class subpopulations over time on phenol and 1-octanol at three different substrate concentrations, compared to a no-added carbon control (*no C*), as indicated. Error bars indicate the calculated standard deviation from the mean in biological triplicates. Subpopulations were taken as the predicted classification with the ANN-32 standard classifier. Classes are colored according to the color legend.

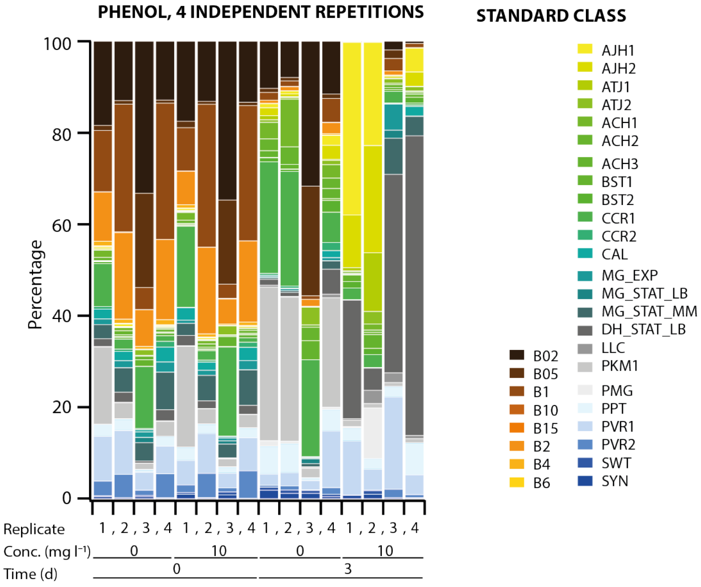

Supplementary figure 6. CellCognize diversity inferred from the ANN-32 classifiers on four independent repetitions of lake water communities enriched with 10 mg l^{-1} phenol. Shown is a stackplot of the predicted classified class abundances, normalized as percentage to the total community size in the respective replicate at each time point (class colors according to legend).

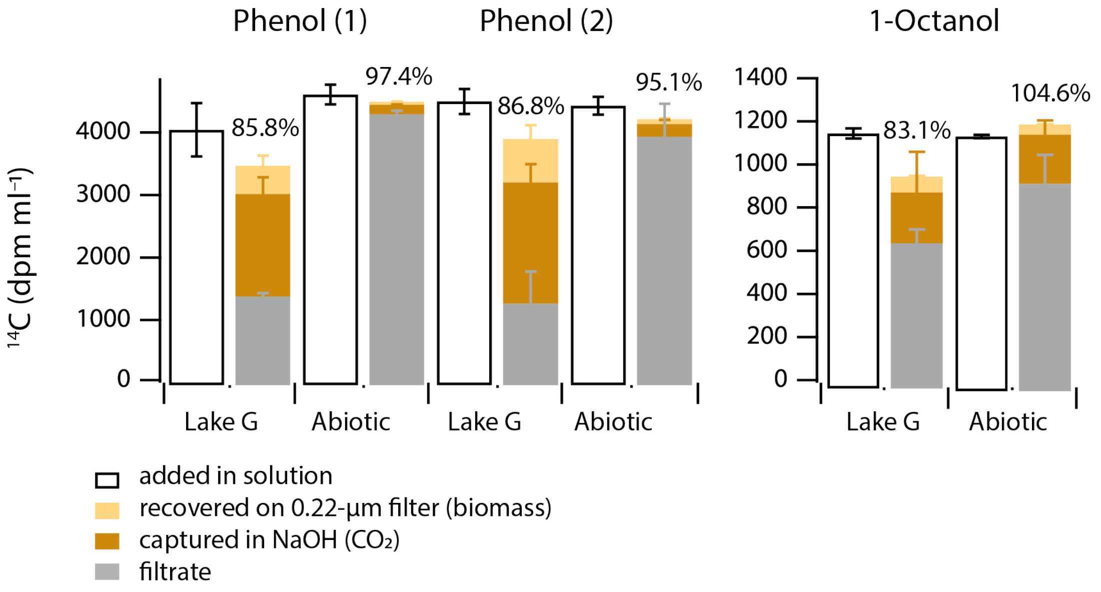

Supplementary figure 7. Mass balance recovery from ^{14}C-labeled substrate experiments. Bars show the mean radioactivity measured after 3 d incubation in two series of phenol and one series of 1-octanol incubations with the Lake Geneva microbiota or abiotic controls without cells (mean ± one SD from biological triplicate experiments). The ^{14}C-substrate was dosed at 4000 (phenol) or 1200 dpm ml^{-1} (1-octanol) amidst 10 mg non-labeled carbon of the same. White bars, radioactivity measured in solution at time 0; ochre bars, radioactivity recovered on 0.22–µm filters and assumed to be the microbial biomass; brown bars, radioactivity recovered in the sodium hydroxide solution after purging at day 3, assumed to consist of dissolved $^{14}C\text{-}CO_2$; grey bars, radioactivity recovered in the filtrate, assumed to consist of remaining non-consumed substrate and dissolved excreted cell material or metabolites. Percentages indicate the mean recovery in the three fractions compared to the original dosed ^{14}C.

Supplementary tables

Supplementary Table 1. Growth conditions of standard strains

**Strain**	**Strain number (own collection)**	**Abbreviation**	**Solid culture medium**	**Liquid culture medium**	**Growth Temperature**
Acinetobacter johnsonii	5045	AJH	Nutrient agar	*Pseudomonas* minimal medium with 10 g l^{-1} succinate	26 °C
Acinetobacter tjernbergiae	5044	ATJ	Nutrient agar	*Pseudomonas* minimal medium with 10 g l^{-1} succinate	26 °C
Arthrobacter chlorophenolicus	2840	ACH	Growth minimal medium	*Arthrobacter* minimal medium, 5 g l^{-1} yeast extract	26 °C
Bacillus subtilis	ATCC 6633	BST	Blood agar	Tryptic soy broth	26 °C
Caulobacter crescentus	2577	CCR	Peptone yeast extract agar	Peptone yeast extract broth	30 °C
Cryptococcus albidus	2632	CAL	Luria-Bertani broth	Luria-Bertani broth	26 °C
Escherichia coli MG1655	4498	MG	Luria-Bertani broth	Luria-Bertani broth, M9-glucose, casamino acids	37 °C
Escherichia coli DH5α-λpir	3044	DH	Luria-Bertani broth	Luria-Bertani broth	37 °C
Lactococcus lactis	1363	LLC	M17 agar	GM17	26 °C
Pseudomonas knackmussii B13	78	PKM	Nutrient agar	*Pseudomonas* minimal medium with 10 g l^{-1} succinate	26 °C
Pseudomonas migulae	5046	PMG	Nutrient agar	*Pseudomonas* minimal medium with 10 g l^{-1} succinate	26 °C
Pseudomonas putida	1291	PPT	Nutrient agar	*Pseudomonas* minimal medium with 10 g l^{-1} succinate	26 °C
Pseudomonas veronii	3370	PVR	Nutrient agar	*Pseudomonas* minimal medium with 10 g l^{-1} succinate	26 °C
Sphingomonas wittichii	2633	SWT	Nutrient agar	*Sphingomonas* minimal medium with 1 g l^{-1} salicylate	30 °C
Sphingomonas	1363	SYN	Nutrient	*Sphingomonas* minimal	30 °C

Strain	Strain number (own collection)	Abbreviation	Solid culture medium	Liquid culture medium	Growth Temperature
yanoikuyae			agar	medium with 1 g l^{-1} salicylate	

Pseudomonas minimal medium: per liter 1 g NH_4Cl, 3.49 g $Na_2HPO_4{\cdot}2H_2O$, 2.77 g KH_2PO_4 at pH 6.8.

Arthrobacter minimal medium: per liter 2.1 g K_2HPO_4, 0.4 g KH_2PO_4, 0.5 g NH_4NO_3, 0.2 g $MgSO_4{\cdot}7H_2O$, 0.023 g $CaCl_2{\cdot}2H_2O$, 2 ml $FeCl_3{\cdot}6H_2O$ solution (1 mg ml^{-1}), pH 7.4.

Sphingomonas minimal medium: per liter 2.44 g Na_2HPO_4, 1.52 g KH_2PO_4, 0.50 g $(NH_4)2SO_4$, 0.2 g $MgSO_4{\cdot}7H_2O$, 0.05 g $CaCl_2{\cdot}2\ H_2O$, 10 ml trace metal solution (0.5 g l^{-1} EDTA, 0.2 g l^{-1} $FeSO_4{\cdot}7H_2O$), 2 ml trace metal solution (per liter 0.1 g $ZnSO_4{\cdot}7H_2O$, 0.03 g $MnCl_2{\cdot}4H_2O$, 0.3 g H_3BO_3, 0.2 g $CoCl_2{\cdot}6H_2O$, 0.01 g $CuCl_2{\cdot}2H_2O$, 0.02 g $NiCl_2{\cdot}6H_2O$, 0.03 g $Na_2MoO_4{\cdot}2H_2O$) at pH 6.9.

Supplementary Table 2. Estimated biovolume and carbon dry mass of the strain and bead standards

**Standard**	**Abbreviation**	**Biovolume (μm^3)**	**Biomass (fg C)**
Acinetobacter johnsonii	AJH	0.32 ± 0.06	82 ± 13
Acinetobacter tjernbergiae	ATJ	0.38 ± 0.04	95 ± 9
Arthrobacter chlorophenolicus	ACH	0.13 ± 0.05	38 ± 12
Bacillus subtilis	BST	0.49 ± 0.28	118 ± 56
Caulobacter crescentus	CCR	0.53 ± 0.09	126 ± 18
Cryptococcus albidus	CAL	5.89 ± 0.05	999 ± 7
Escherichia coli DH5α-λpir	DH	0.37 ± 0.05	92 ± 11
Lactococcus lactis	LLC	0.40 ± 0.05	99 ± 11
Pseudomonas knackmussii	PKM	0.23 ± 0.05	61 ± 11
Pseudomonas migulae	PMG	0.39 ± 0.08	97 ± 17
Pseudomonas putida	PPT	0.30 ± 0.06	77 ± 13
Pseudomonas veronii	PVR	0.76 ± 0.23	172 ± 44
Sphingomonas wittichii	SWT	0.30 ± 0.16	77 ± 34
Sphingomonas yanoikuyae	SYN	0.24 ± 0.03	64 ± 7
0.2 μm bead	B02	0.12 ± 0.04	35 ± 10
0.5 μm bead	B05	0.46 ± 0.31	112 ± 62
1 μm bead	B1	0.60 ± 0.40	140 ± 77
2 μm bead	B2	2.0 ± 1.0	395 ± 165
4 μm bead	B4	3.0 ± 1.0	559 ± 157
6 μm bead	B6	7.0 ± 1.0	1159 ± 141
10 μm bead	B10	21 ± 5.0	2982 ± 601
15 μm bead	B15	53 ± 2.0	6612 ± 214

Mean biovolume ± one SD measured from holographic microscopy of beads or cells (5 images per culture with 25-50 objects each), growing under the standardized conditions of Supplementary Table 1 until stationary phase. No subpopulations were distinguished in the biovolume measurements. Mean biomass inferred from $m_b = 435V^{0.86}$; with m_b the calculated dry weight of the cell, and V the measured biovolume; we further assumed a 50% C-content [2].

Supplementary notes: Glossary of terms

Anchoring: Adding two artificial data points to the filtered, gated and log-transformed FCM dataset before scaling in the Neural network procedure for training or for classifier prediction, in order to 'fix' the dataset in multidimensional space. The anchoring points consist of the log-transformed values of the set thresholds.

Cell type diversity: Predicted diversity in a microbial community based on classification to a predefined set of standards.

Classes: Used output attributions from the Neural network training and classifications; typically the same number and names as the used standards.

Classification: The attribution or assignment of cells or events in a dataset to each of the output classes, for example, based on their maximum probability or similarity score.

Classifier: Learned linear equation produced by the training, validation and testing of the Neural network, used to calculate the probability of each cell or event in a dataset to be attributed to each of the output classes.

Correct predicted classification: The number of cells in a dataset with known composition attributed (assigned) to their actual output class(es) based on their maximum individual probability score. Can be expressed as percentage of the intended number of added cells.

Filtering: The removal of noise or spurious (negative) data points from an FCM data set, comprising values below or above defined thresholds, for each of the FCM channels.

Gating: Defining the closest coherent (sub)population(s) on an FCM dataset from an individual standard for the classification, and restricting events from the dataset to that definition.

Mean classification similarity score: The mean probability score of the cells in a dataset attributed to a particular output class, divided by the mean probability score of the cells corresponding to the used standard for that output class, attributed to their own output class.

Precision: The number of true positives divided by the sum of true and false positives in a dataset, expressed as percentage.

Predicted classification: The number of cells in a dataset with unknown composition attributed (assigned) to one or more of the defined output classes based on their highest individual probability score. Can be expressed as percentage of all attributed cells.

Recall: The number of true positives divided by the sum of true positives and false negatives in a dataset, expressed as percentage.

Standards: Used filtered and gated coherent sets of input reference data sets, for example consisting of defined beads or microbial pure cultures grown under defined conditions.

Supplementary Methods

Relevant files can be found on : Zenodo.org; DOI: 10.5281/zenodo.3822094

Section 1. Data pretreatment

FCM data were filtered for each of the seven parameters between a fixed lower (generally a value of 100) and an upper boundary (10^5–10^7), and then $\log_{10}$-transformed. Filtered and log-transformed data for each of the standards were plotted in 2D combinations of FITC-H, SSC-H and FSC-H to identify potential subpopulations (see, e.g., Fig. S1). Subpopulations containing at least 5% of all data were gated and separated within the filtered data sets by setting lower and upper log-transformed boundaries in each of the three-parameter dimensions (i.e., FITC-H, SSC-H and FSC-H). This process resulted in a total of 32 standards: 8 bead and 24 strain data sets (see Table 1). For the preliminary experiment with three strains (see below), we used five standards (three strains, two of which had two subpopulations).

1.1 Filtering of FCM data

```
% path: Files_for_Zenodo/FCM_files
load('final_file_merged_2019.mat');
```

This file has the combined FCM data of the 22 used standards (8 beads, 14 microbial pure cultures) as in Table S2. Order of the data is:

```
Merged 22
1=B02
2=B05
3=B1
4=B10
5=B15
6=B2
7=B4
8=B6

9=AJH
10=ATJ
11=ACH
12=BST
13=CCR
14=CAL
15=ECL
16=LLC
17=PKM
18=PMG
19=PPT
20=PVR
21=SWT
22=SYN
```

Filter the files to within the lower and upper boundary **thresholds**, for each of the seven FCM channels. Order of the FCM channels in the files is:

```
Column 1= FSC-H
Column 2= SSC-H
Column 3= FITC-H
Column 4= FSC-A
Column 5= SSC-A
Column 6= FITC-A
Column 7= Width
```

Define the min and maxdata values for the filtering.

```
mindata1=100;
maxdata1=4000000;
mindata2=100;
maxdata2=4000000;
mindata3=100;
maxdata3=500000;
mindata4=100;
maxdata4=2000000;
```

```
mindata5=100;
maxdata5=2000000;
mindata6=100;
maxdata6=1000000;
mindata7=10;
maxdata7=2000;
```

```
for i=1:22
final_files1=final_files{i};

final_files1_filtered=final_files1(final_files1(:,1)<maxdata1,:);
final_files1_filtered=final_files1_filtered(final_files1_filtered(:,1)>mindata1,:);
final_files1_filtered=final_files1_filtered(final_files1_filtered(:,2)<maxdata2,:);
final_files1_filtered=final_files1_filtered(final_files1_filtered(:,2)>mindata2,:);
final_files1_filtered=final_files1_filtered(final_files1_filtered(:,3)<maxdata3,:);
final_files1_filtered=final_files1_filtered(final_files1_filtered(:,3)>mindata3,:);
final_files1_filtered=final_files1_filtered(final_files1_filtered(:,4)<maxdata4,:);
final_files1_filtered=final_files1_filtered(final_files1_filtered(:,4)>mindata4,:);
final_files1_filtered=final_files1_filtered(final_files1_filtered(:,5)<maxdata5,:);
final_files1_filtered=final_files1_filtered(final_files1_filtered(:,5)>mindata5,:);
final_files1_filtered=final_files1_filtered(final_files1_filtered(:,6)<maxdata6,:);
final_files1_filtered=final_files1_filtered(final_files1_filtered(:,6)>mindata6,:);
final_files1_filtered=final_files1_filtered(final_files1_filtered(:,7)<maxdata7,:);
final_files1_filtered=final_files1_filtered(final_files1_filtered(:,7)>mindata7,:);
```

Do the log10-transformation for each column specifically.

```
scales1=log10(final_files1_filtered(:,1));
scales2=log10(final_files1_filtered(:,2));
scales3=log10(final_files1_filtered(:,3));
scales4=log10(final_files1_filtered(:,4));
scales5=log10(final_files1_filtered(:,5));
scales6=log10(final_files1_filtered(:,6));
scales7=log10(final_files1_filtered(:,7));
```

Regroup the columns back into one file.

```
scales=horzcat(scales1,scales2,scales3,scales4,scales5,scales6,scales7);
standard{i}=scales;
end;
```

1.2 Gating

Gate the different standards to either individual or multiple subpopulations, depending on the 2D plot aspect of FCS-H vs SSC-H vs FITC-H (example in Fig. S1). Bead standards do not require further gating.

```
%Rename the bead standards

B02=standard{1};
B05=standard{2};
B1=standard{3};
B10=standard{4};
B15=standard{5};
B2=standard{6};
B4=standard{7};
B6=standard{8};

%gating AJH1, example

S9=((standard{9}(:,1)>3.8) & (standard{9}(:,1)<4.8) & (standard{9}(:,2)>2.5) &
(standard{9}(:,2)< 3.3) & (standard{9}(:,3)>4.5) & (standard{9}(:,3)<5));
S9=repelem(S9,1,7);
AJH1=standard{9}(S9);
AJH1=reshape(AJH1,[],7);

%gating AJH2

S9=((standard{9}(:,1)>3.8) & (standard{9}(:,1)<4.8) & (standard{9}(:,2)>3.2) &
(standard{9}(:,2)< 4.0) & (standard{9}(:,3)>5) & (standard{9}(:,3)<5.5));
S9=repelem(S9,1,7);
AJH2=standard{9}(S9);
AJH2=reshape(AJH2,[],7);

%gate all others, as desired. Complete gated output file, see below.
```

Add the four separate *E. coli* standards from exponential and stationary phase, and grown on different media (see Section 3.5)

```
%path: /Files_for_Zenodo/FCM_files
load('ALL_ECOLI_LOG.mat');
%has log-transformed data
ECL_ALL={ECL_EXP,ECL_STAT_LB,ECL_STAT_MM, ECL};
```

```
% gate the Ecoli files, as desired, for example ECL_EXP1

S1=((standard{1}(:,1)>3.0) & (standard{1}(:,1)<4.0) & (standard{1}(:,2)>3.0) &
(standard{1}(:,2)< 3.6) & (standard{1}(:,3)>3.2) & (standard{1}(:,3)<4.4));
S1=repelem(S1,1,7);
ECL_EXP1=standard{1}(S1);
ECL_EXP1=reshape(ECL_EXP1,[],7);

% repeat for all, if necessary
```

The ECL subpopulations 1 and 2 in exponential growth conditions are not further included, because their relative abundance was below 5%.

Now combine the filtered gated standard files into one. This is the 32-standard input file for the ANN model.

```
filtered_standards={B02,B05,B1,B10,B15,B2,B4,B6,AJH1,AJH2,ATJ1,ATJ2,ACH1,ACH2,ACH3,BST1,BST2,
CCR1,CCR2,CAL,ECL_EXP3,ECL_STAT_LB,ECL_STAT_MM,ECL,LLC,PKM1,PMG,PPT,PVR1,PVR2,SWT,SYN};

%file saved as 'filtered_standards_32.mat'
```

Section 2. Artificial neural network reconstruction.

The filtered and gated data sets of the standards ($n \sim 3 \times 10^5$ to 1.5×10^6 events per standard) were used as input for the development of ANN models. The datasets were randomly subsampled to 10,000 events per standard using the *datasample* function (Matlab v. R2017a). Crucially, the lower and upper boundary thresholds imposed during the filtering process for each of the seven FCM parameters were added as two data points ("anchors") per parameter to the first subsampled standard.

Subsampled anchored datasets were concatenated and used as input into the ANN model, during which they were further scaled (between −1 and 1, hence the necessity to add the anchors) and randomly divided using *Dividerand* (Matlab v. R2017a) into three blocks: a training set (50% of the data), a validation set (25%) and a testing set (25%).

The ANN architecture consisted of a feed-forward back-propagation algorithm with one input, one hidden and one output layer. The input layer contained 7 nodes (corresponding to the 7 FCM parameters), whereas the output layer contained 5 (for the preliminary three-strain experiment) or 32 nodes (one for each of the standards in the full set). Input nodes were connected to the hidden layer by the *sigmoid* function (Matlab v. 2017a), whereas the hidden layer nodes (20) were connected to the output by the *softmax* transfer function (Matlab v. 2017a). The input matrix was trained using the *trainscg* function (Matlab v. 2017a) in a 1000-cycle of training, validation and testing (performance goal = 0 | time = Inf | min grad = 10^{-6} | max fail = 6). Performance of the ANN was evaluated by *crossentropy*. The outcome of the ANN model is a learned linear equation, termed the ANN classifier, describing the correlations between input parameters and the five (proof-of-concept experiment, ANN-5) or 32 classes of the standard dataset (ANN-32). The process of subsampling, anchoring, concatenation and training was repeated five times independently on the full datasets, generating five slightly different ANN classifiers. The performance of the ANN classifiers was assessed on the basis of confusion matrices (Matlab v. 2017a), representing predicted versus actual events for the complete *in silico* mixed set of standards, and the false prediction rate (as shown in Fig. S2).

2.1. Subsampling and anchoring

Data were first randomly subsampled to same number of events. File array name from previous scaling was 'filtered_standards'.

```
%path /Files_for_Zenodo/FCM_files
load 'filtered_standards_32.mat'

standard_normz=[];
sample_size=20000;
        for i=1:length(filtered_standards)
                standard_normz{i,1}=datasample(filtered_standards{1,i},sample_size,1);
        end;
standard_normz=standard_normz';

%alternatively: subsample to n = 5000. This file is saved as
'standard_normz_restricted_32.mat' for section 3.7
```

Add the line with the anchors for proper and consistent scaling throughout all data sets.

```
anchors=[2,2,2,2,2,2,1;6.6,6.6,5.7,6.3,6.3,6.0,3.3];

standard_normz{1}=vertcat(anchors,standard_normz{1});
```

2.2. ANN selection, training and validation

Continue ANN with subsampled data set (standard_normz); here 20000 events per standard.

```
file_length_final=cellfun(@length, standard_normz);
file_size=file_length_final;
file_length_final=[1 file_length_final];
file_length_final=cumsum(file_length_final);
input=vertcat(standard_normz{:});
output=zeros(length(input),length(standard_normz));
for i=1:length(file_length_final)-1
output(file_length_final(i):file_length_final(i+1)-1,i)=1;
end

input=input';
output=output';


x = input;
t = output;
```

Choose a Training Function

```
trainFcn = 'trainscg';  % Scaled conjugate gradient backpropagation.
```

Create a Pattern Recognition Network

```
hiddenLayerSize = 20;
net = patternnet(hiddenLayerSize);
```

Setup Division of Data for Training, Validation, Testing

```
net.divideFcn = 'dividerand';  % Divide data randomly
net.divideMode = 'sample';  % Divide up every sample
net.divideParam.trainRatio = 50/100;
net.divideParam.valRatio = 25/100;
net.divideParam.testRatio = 25/100;
```

Choose a Performance Function

For a list of all performance functions type: help nnperformance

```
net.performFcn = 'crossentropy';  % Cross-Entropy
```

Choose Plot Functions

```
net.plotFcns = {'plotperform','plottrainstate','ploterrhist', ...
    'plotconfusion', 'plotroc'};
```

Train the Network

```
[net,tr] = train(net,x,t);
```

Test the Network

```
y = net(x);
e = gsubtract(t,y);
performance = perform(net,t,y);
tind = vec2ind(t);
yind = vec2ind(y);
percentErrors = sum(tind ~= yind)/numel(tind);
```

Recalculate Training, Validation and Test Performance

```
trainTargets = t .* tr.trainMask{1};
valTargets = t .* tr.valMask{1};
testTargets = t .* tr.testMask{1};
trainPerformance = perform(net,trainTargets,y)
valPerformance = perform(net,valTargets,y)
testPerformance = perform(net,testTargets,y)

figure, plotconfusion(t,y)

genFunction(net,'GIVE_FILE_NAME');
```

The section 2.1 and 2.2 process was repeated five times starting from the random subsampling, to generate five independent NN-classifier functions.

Example classifier
/Files_for_Zenodo/NN_file_example/NNfunction_wide_anchor_normz_filtered_260619.m

2.3 Analysis of Fig. 2c, confusion plot

The confusion plot of **Fig. 2c** is a heatmap of the mean from those five trainings (data as reported in Dataset 1), each with 10,000 subsampled data. Precision, the number of true positives divided by the sum of true and false positives in a dataset, is shown as rows in the confusion plot. Recall, the number of true positives divided by the sum of true positives and false negatives in a dataset, is shown as columns. The means of those are reported in Table 1.

Section 3. CellCognize testing of standard-mixed communities.

In a first proof-of-concept experiment, we cultured *E. coli* MG1655, *P. veronii* and *A. johnsonii* individually to stationary phase, diluted cultures 1:1000 in PBS, and measured cells by FCM after staining with Sybr Green I either individually, or in different mixtures of all three strains combined. Individual and mixture data sets were analyzed with CellCognize using a set of five replicate ANN-5 classifiers, comparing expected added cell numbers of each of the three strains with their assigned class attributions from the ANN-5 classifiers.

3.1 Preparing a limited ANN with five standards only.

```
% data import. Read filtered, log-transformed and gated E. coli, P. veronii and A. johnsonii
data files. This has five datasets

% path: /Files_for_Zenodo/FCM_files/MIX_experiment_ACL_AJH_PVR/
filtered_standards_AJH_ECL_PVE.mat

filtered_standards={AJH1,ECL1,ECL2,PVR1,PVR2};
```

Continue training, validating and testing ANN-5 with subsampled data set; here 5000 events per sample; as in sections 2.1 and 2.2

Example output classifier file saved as /Files_for_Zenodo/NN_file_example/NNfunction_wide_anchor_normz_filtered_trimix_310819.m

3.2 Analyze the synthetic community mixtures.

```
%Read in and treat data files of either cultures alone, or in combinations. Same folder:
/Files_for_Zenodo/FCM_files/MIX_experiment_ACL_AJH_PVR
%as example: combination of 30/10/10 ECL/AJH/PVE

LWECO1=readtable('Specimen2_A6_EAP30-10-10-1.csv');
LWECO1=table2array(LWECO1);
LWECO2=readtable('Specimen2_B6_EAP30-10-10-2.csv');
LWECO2=table2array(LWECO2);
LWECO3=readtable('Specimen2_C6_EAP30-10-10-3.csv');
LWECO3=table2array(LWECO3);
LWECO4=readtable('Specimen2_D6_EAP30-10-10-4.csv');
LWECO4=table2array(LWECO4);

community_combined=vertcat(LWECO1,LWECO2,LWECO3,LWECO4);

%has 8 columns, remove column 8

community_combined(:,8)=[];

%filtering and log-transformation as in section 1.1

%regroup the columns back into one file

input_community=horzcat(scales1,scales2,scales3,scales4,scales5,scales6,scales7);

%add anchor line

anchors=[2,2,2,2,2,2,1;6.6,6.6,5.7,6.3,6.3,6.0,3.3];

input_community=vertcat(anchors,input_community);

%transpose numeric array to be conform to NN input

input_community=input_community';

% run NN functions without thresholding
```

```
% vec2ind means that the index of the row is find where the value of 1 occurs. In this table
it is the value that is rounded up to 1!
% NN functions are in switchdrive/final_files/Community_diversity_analysis/NN functions

%make a table with the classes (1-5), a '0' (for the non-classified) - and one extra (33)
that works as an anchor to fill the list properly (…)

classes=(0:6);
empty_class=[6;0];

%NN function (1)

output_restricted_anchor1=
NNfunction_wide_anchor_normz_filtered_trimix_310819(input_community); %available in example
output_restricted_anchor_final1=vec2ind(output_restricted_anchor1);

membership_restricted1 =
histc(output_restricted_anchor_final1,unique(output_restricted_anchor_final1));

classes_restricted1=unique(output_restricted_anchor_final1);
final_community1= [classes_restricted1;membership_restricted1];

%now add the empty class to the final community

finalComm1=[final_community1,empty_class];

%apply a logical function to find corresponding values in the list with all categories in the
file 'classes'

[lic,loc]=ismember(classes,finalComm1(1,:));
results1(2,lic)=finalComm1(2,loc(lic));
results1(1,:)=classes;

%produce final summary community and save as .csv. Modify the path if necessary.

final_results=vertcat(results1);

T=array2table(final_results','VariableNames',{'Class1','Count1'});

%write results to table, as example:

writetable(T,'FILE_NAME.csv');
```

3.3. Analysis of Figure 2a.

Four replicates of each strain individually diluted were measured on FCM and this datafile was then analyzed by the ANN-5 classifier. Output categories were summed and the proportion of the correct classification prediction was calculated. For example, the percent correct classification of pure E. coli by the ANN-5 was 86% (=ECL1 + ECL2 in Table 3.3.1).

Table 3.3.1: Example output and recovery calculation from the ANN-5 classifier for the pure culture E. coli data set.

ANN5_Class attribution	Class	Count in class	Mean Class Assignment Probability	Percent Recovery
1	AJH	419	0.369205176	0.01
2	ECL1	10531	0.750591042	0.23
3	ECL2	28760	0.82239543	0.63
4	PVR1	2289	0.329680958	0.05
5	PVR2	3559	0.395254884	0.08
sum		45558		

Four different mixtures were prepared of the three strain suspensions. These were again measured on four individual replicates, which were combined and classified with the ANN-5 classifier. These attributions were then compared to the actual expected cell numbers measured from the individual strains multiplied by the dilution factors.

Table 3.3.2: Actual and expected cell attributions in synthetic mixtures of three strains.

Class	Percent of total AJH alone	Percent of total ECL alone	Percent of total PVR alone	Percent of total EAP10-10-10	Percent expected	Percent of total EAP10-10-30	Percent expected	Percent of total EAP10-30-10	Percent expected	Percent of total EAP30-10-10	Percent expected
AJH	87.67%	0.92%	11.31%	42.56%	113.7	31.37%	104.5	61.91%	92.7	22.82%	101.6
ECL1	0.20%	23.12%	3.50%	9.29%	95.7[a]	8.63%	109.0[a]	5.32%	91.7[a]	15.39%	86.5[a]
ECL2	1.13%	63.13%	9.77%	25.87%		23.47%		14.70%		41.74%	
PVR1	10.51%	5.02%	9.68%	9.49%	116.1[a]	11.99%	79.2[a]	11.03%	158.5[a]	8.90%	174.4[a]
PVR2	0.48%	7.81%	65.74%	12.79%		24.53%		7.04%		11.16%	
#cells	46446	45558	23793	124026		154725		231478		226426	
mixture	10 µl AJH in 1 ml	10 µl ECL in 1 ml	10 µl PVR in 1 ml	10 µl ECL<br>10 µl AJH<br>10 µl PVR<br>in 1 ml		10 µl ECL<br>10 µl AJH<br>30 µl PVR<br>in 1 ml		10 µl ECL<br>30 µl AJH<br>10 µl PVR<br>in 1 ml		30 µl ECL<br>10 µl AJH<br>10 µl PVR<br>in 1 ml	

a)ECL = ECL1+ECL2, PVR=PVR1+PVR2

3.4 PCA analysis of Figure 2b.

For PCA analysis, we used the filtered and gated data set of the 32 standards and their subpopulations (from section 1.2 above). This was transformed into a single matrix, with columns being the different standards, and rows being the concatenated 7 flow cytometry variables (subsampled for n = 20 000), one underneath each other (FSC-H, SSC-H, FITC-H, FSC-A, SSC-A, FITC-A and width).

```
%take the file standard_normz from section 1.2
% remove the anchoring lines in cell 1

standard_normz{1}(1:2,:)=[];

for i=1:32;
strain=vertcat(standard_normz{i}(:,1),standard_normz{i}(:,2),standard_normz{i}(:,3),standard_
normz{i}(:,4),standard_normz{i}(:,5),standard_normz{i}(:,6),standard_normz{i}(:,7));
if i==1;
strainm=strain;
strain=[];
else
strainm=horzcat(strainm,strain);
strain=[];
end
end;

%strainm is now a 140 000 x 32 double


%doing regular PCA

[coeff,score,latent,tsquared,explained,mu]=pca(strainm);
fig=scatter(coeff(:,1),coeff(:,2));

%save output file 'GIVE_NAME.pdf'

Explained:

First dimension:      69.1327
Second dimension:     26.3605
```

```
Third dimension:       1.5639
```

3.5 Analysis of Figure 2d.

In order to evaluate whether CellCognize could distinguish different cell physiologies, we classified FCM datasets of all four *E. coli* standards (representing different strains, culture media, and cell growth phases) individually (randomly subsampled to n = 20,000 cells) or as an *in silico* mixture with n = 5000 cells of each, using the five ANN-32 classifiers.

Individual class assignments:

E. coli MG1655 was cultured independently on LB or M9-CAA medium to exponential phase (OD = 0.5) and to stationary phase (OD = 2). Cultures were diluted in artificial lake water at 10^{-4}, 10^{-5} , and 10^{-6} , stained and individually measured in two technical replicates on FCM. Data were extracted, filtered, log-transformed and anchored as described above, and analyzed with the five ANN-32 classifiers for standard class attributions.

Filtering, gating and log-transforming the datasets:

```
% Prepare Eco DH5a standard (ECL), filtering, log-transformation and gating as in Sections
1.1 and 1.2

%final file has four Ecoli standards
% 1 = exponential phase MM, for MG1655
% 2 = Stationary phase LB, for MG1655
% 3 = Stationary phase MM, for MG1655
% 4 = Stationary phase LB, for DH5alpha

ECL_ALL={ECL_EXP,ECL_STAT_LB,ECL_STAT_MM,ECL};

% file saved as ALL_ECOLI_LOG.mat
```

3.6 Run ANN-32 classifications

```
%for each of the four individual datasets

load('ALL_ECOLI_LOG.mat')

input_community=ECL_ALL{4};

%add anchor line

anchors=[2,2,2,2,2,2,1;6.6,6.6,5.7,6.3,6.3,6.0,3.3];

input_community=vertcat(anchors,input_community);

%transpose numeric array to be conform to NN input

input_community=input_community';

% run NN functions without thresholding
% vec2ind means that the index of the row is find where the value of 1 occurs. In this table
it is the value that is rounded up to 1!
% change path to the folder with the NN functions are in /NN_file_example

%make a table with the classes (1-32), a '0' (for the non-classified) - and one extra (33)
that works as an anchor to fill the list properly (…)

classes=(0:33);
empty_class=[33;0];

%NN function (1)

output_restricted_anchor1= NNfunction_wide_anchor_normz_filtered_260619(input_community);
output_restricted_anchor_final1=vec2ind(output_restricted_anchor1);

membership_restricted1 =
histc(output_restricted_anchor_final1,unique(output_restricted_anchor_final1));

classes_restricted1=unique(output_restricted_anchor_final1);
final_community1= [classes_restricted1;membership_restricted1];
```

```
%now add the empty class to the final community

finalComm1=[final_community1,empty_class];

%apply a logical function to find corresponding values in the list with all categories in the file 'classes'

[lic,loc]=ismember(classes,finalComm1(1,:));
results1(2,lic)=finalComm1(2,loc(lic));
results1(1,:)=classes;

% run more NN functions as desired or available

%produce a final summary community and save as .csv.


final_results=vertcat(results1);

T=array2table(final_results','VariableNames',{'Class1','Count1'});

%write results to table.

writetable(T,'output.csv');
```

3.7 In-silico mixture of four E. coli strains with lake water background.

```
%%%Lake water FCM files

load('lakewaterlinear.mat')
% is combined file of Specimen1_E1 to Specimen1_E4 in the same folder

%has 7 columns

%do the filtering and log-transformations as in section 1.1

%regroup the columns back into one file

input_community=horzcat(scales1,scales2,scales3,scales4,scales5,scales6,scales7);

%% change path. Retrieve the four E. coli filtered and gated standards.

load('standard_normz_restricted_32.mat')

%This file has the filtered and gated, subsampled n = 5000 standard sets. Without that file, take the one that is the output of section 2.1.

MG_EXP3=standard_normz{21};
MG_STAT_LB=standard_normz{22};
MG_STAT_MM=standard_normz{23};
DH=standard_normz{24};

anchors=[2,2,2,2,2,2,1;6.6,6.6,5.7,6.3,6.3,6.0,3.3];

input_community=vertcat(anchors,MG_EXP3,MG_STAT_LB,MG_STAT_MM,DH,input_community);

input_community=input_community';

% change to path with example NN functions /NN_file_example

%make a table with the classes (1-32), a '0' (for the non-classified) - and one extra (33) that works as an anchor to fill the list properly (…)

classes=(0:33);
empty_class=[33;0];

%NN function (1)

output_restricted_anchor1= NNfunction_wide_anchor_normz_filtered_260619(input_community);
max_probability=max(output_restricted_anchor1);
output_restricted_anchor_final1=vec2ind(output_restricted_anchor1);
```

```
membership_restricted1 =
histc(output_restricted_anchor_final1,unique(output_restricted_anchor_final1));

classes_restricted1=unique(output_restricted_anchor_final1);
final_community1= [classes_restricted1;membership_restricted1];

%now add the empty class to the final community

finalComm1=[final_community1,empty_class];

%apply a logical function to find corresponding values in the list with all categories in the
file 'classes'

[lic,loc]=ismember(classes,finalComm1(1,:));
results1(2,lic)=finalComm1(2,loc(lic));
results1(1,:)=classes;

for i=1:25038;
               A=output_restricted_anchor1(:,i);
               C=max_probability(:,i);
               A(A<C)=0;
               output_restricted_anchor1(:,i)=A;
end

prop_MG_EXP3_1=numel(nonzeros(output_restricted_anchor1(21,(2:5002))));
prop_MG_STAT_LB_1=numel(nonzeros(output_restricted_anchor1(22,(5003:10003))));
prop_MG_STAT_MM_1=numel(nonzeros(output_restricted_anchor1(23,(10003:15003))));
prop_DH_1=numel(nonzeros(output_restricted_anchor1(24,(15003:20003))));
prop1=[prop_MG_EXP3_1,prop_MG_STAT_LB_1,prop_MG_STAT_MM_1,prop_DH_1];

% run more NN functions as desired o as available


%produce final summary community and save as .csv.


final_results=vertcat(results1);

T=array2table(final_results','VariableNames',{'Class1','Count1'});

final_proportions=vertcat(prop1);

writetable(T,'OUTPUT.csv');
```

Calculate the mean assigned proportions to the *E. coli* classes (in the table T) and compare to the mean of the true (known) values of their final proportions.

3.8 Analysis of Figure 2e: Class attribution of aquatic microbial community.

An aquatic microbial community from Lake Geneva was recovered from 2 L of lake water, sampled at 1 m depth at a site close to the shore in Saint-Sulpice (46.517°N, 6.579°E), and used as an unknown background microbial community. Debris was removed by filtering the lake water through a nylon cell strainer with 40-µm pore size (Falcon, USA). Bacterial cells were then collected from the filtrate using a 0.2-µm pore size polyethersulfone membrane filter (Sartorius, Switzerland). The filter with the cells was resuspended during 2 h in artificial lake water mineral medium (ALW; containing, per L, 36.4 mg $CaCl_2{\cdot}2H_2O$, 0.25 mg $FeCl_3{\cdot}6H_2O$, 112.5 mg $MgSO_4{\cdot}7H_2O$, 43.5 mg K_2HPO_4, 17 mg KH_2PO_4, 33.4 mg $Na_2HPO_4{\cdot}2H_2O$, and 25 mg NH_4NO_3). Cell density in the ALW microbial suspension was then quantified and diluted to 10^5 cells per ml. The diluted samples were stained with SYBR Green I for 30 min in the dark, and then measured in FCM, in three biological replicates, each with two technical replicates. FCM data were exported as .csv format, merged, filtered between lower and upper boundaries, and log-transformed for each of the seven FCM parameters as described above. The same two (low and high) anchor values per FCM parameter were then added to the dataset to ensure its proper ‘positioning’ during the ANN classifier computation. The lake water microbial community data were analyzed alone (n = 5039 cells), and also after being merged *in silico* with each of the 32 standards individually, randomly subsampled (n = 5000) for that purpose.

Datasets were then classified using each of the five ANN-32 classifiers, in order to attribute all events to the predefined standard classes. In a further test, randomly subsampled FCM datasets (*n* = 5000) of three standards each (AJH1, MG_STAT_MM and PVR1) were merged *in silico* with the lake water community (*n* = 5039 cells) and reclassified using the ANN-32 classifiers. The recovery rate was calculated as the ratio of the number of cells from the standard attributed to its own class and the *in silico* added number. The mean probability and probability distribution of attribution were calculated for those particles assigned to each class (for example, in Fig. 4b).

```
%read in Lakewater data sets

load('lakewaterlinear.mat')

%do the filtering and log transformation as in section 1.1

%regroup the columns back into one file

input_community=horzcat(scales1,scales2,scales3,scales4,scales5,scales6,scales7);

% add anchor line

anchors=[2,2,2,2,2,2,1;6.6,6.6,5.7,6.3,6.3,6.0,3.3];

input_community=vertcat(anchors,input_community);

%transpose numeric array to be conform to NN input

input_community=input_community';
```

Follow the ANN-32 classification as in section 3.6 to produce an output table with the class assignments. Calculate mean and standard deviation as in Fig. 2a, top panel.

3.9 In silico mixing three standards into lake water and back-tracing. Fig. 2e lower panel.

```
%%%

load('lakewaterlinear.mat')

%has 7 columns

%do the filtering and log transformation as in Section 1.1

%regroup the columns back into one file

input_community=horzcat(scales1,scales2,scales3,scales4,scales5,scales6,scales7);

%%Load the files of the filtered, gated, log-transformed and subsampled pure culture
standards.
%%

load('standard_normz_restricted_32.mat')

% from section 2.1; this has a set of subsampled n = 5000 individual standards

AJH1=standard_normz{9};
ECL=standard_normz{23};
PVR1=standard_normz{29};

%add anchors and mix with lakewater community into a single file

anchors=[2,2,2,2,2,2,1;6.6,6.6,5.7,6.3,6.3,6.0,3.3];

input_community=vertcat(anchors,AJH1,ECL,PVR1,input_community);

input_community=input_community';

% NN example function in /NN_file_example

%make a table with the classes (1-32), a '0' (for the non-classified) - and one extra (33)
that works as an anchor to fill the list properly (…)
```

```
classes=(0:33);
empty_class=[33;0];

%NN function (1)

output_restricted_anchor1= NNfunction_wide_anchor_normz_filtered_260619(input_community);
max_probability=max(output_restricted_anchor1);
output_restricted_anchor_final1=vec2ind(output_restricted_anchor1);

membership_restricted1 =
histc(output_restricted_anchor_final1,unique(output_restricted_anchor_final1));

classes_restricted1=unique(output_restricted_anchor_final1);
final_community1= [classes_restricted1;membership_restricted1];

%now add the empty class to the final community

finalComm1=[final_community1,empty_class];

%apply a logical function to find corresponding values in the list with all categories in the
file 'classes'

[lic,loc]=ismember(classes,finalComm1(1,:));
results1(2,lic)=finalComm1(2,loc(lic));
results1(1,:)=classes;

for i=1:20038;
               A=output_restricted_anchor1(:,i);
               C=max_probability(:,i);
               A(A<C)=0;
               output_restricted_anchor1(:,i)=A;
end

prop_AJH_1=numel(nonzeros(output_restricted_anchor1(9,(2:5002))));
prop_ECL_1=numel(nonzeros(output_restricted_anchor1(23,(5003:10003))));
prop_PVR_1=numel(nonzeros(output_restricted_anchor1(29,(10003:15003))));
prop1=[prop_AJH_1,prop_ECL_1,prop_PVR_1];

% run more NN functions as desired or as available

% produce final summary community and save as .csv.


final_results=vertcat(results1);

T=array2table(final_results','VariableNames',{'Class1','Count1'});

final_proportions=vertcat(prop1);

writetable(T,'OUTPUT.csv');
```

Recovery rates were calculated as the mean percentage of each standard attributed to its own class versus the true added numbers.

3.10 Fig. 2f Analysis.

The performance of ANN-32 classifiers was further evaluated by mixing stationary phase-grown *E. coli* into the filtered lake water microbial community samples. *E. coli* MG1655 was cultured either on LB or on M9-CAA. Cells were counted in stationary phase samples, diluted in artificial lake water, and added as 1.0×10^4 or 1.0×10^5 cells ml^{-1} to the lake water community. Mixtures were stained and measured on FCM for comparison with lake water microbial community samples alone.

```
%read in individual FCM files of Ecoli in LW, all dilutions 10e-4, 10e-5, 10e-6; both media
LB and MM separately.
%path: Ecoli_Lakewater/Lake_water_mixed_Ecoli/LB_medium

%Clean and filter data sets to properly count all events. Example:

LWECO1=readtable('100000cells.csv');
LWECO1=table2array(LWECO1);
```

```
LWECO2=readtable('100000cells(1).csv');
LWECO2=table2array(LWECO2);

community_combined=vertcat(LWECO1,LWECO2);

%remove 8th columns

community_combined(:,8)=[];


%do the filtering and log transformation as in section 1.1

%regroup the columns back into one file

input_community=horzcat(scales1,scales2,scales3,scales4,scales5,scales6,scales7);

%%number of elements in this file = 5885
%% repeat for all files
%%Classify all datasets per condition, grouped across replicates, using the ANN-32 classifier
as in Section 3.6 above.

```

Calculate percent recovery as the classified number of cells to the appropriate category divided by the expected number of added cells.

Table 3.10.1: Recovery calculation of regrown E. coli mixed with lake water, classified using ANN-32.

Sample	dilu-tion	#cells	nr files	volume per analysis (ml)	total volume per analysis	#cells per ml	# cells in class ECL	#cells in ECL-class per ml	#cells in ECL per ml minus LW bg	vol added of ECL (ml)	expected #cells per ml added	%re co-very
LW background		5036	4	0.02	0.08	62950	204	2550				
ECL M9_CAA	10^{-4}	14750	1	0.02	0.02	737500						
	10^{-3}	118852	1	0.02	0.02	5942600						
	10^{-2}	860521	1	0.02	0.02	43026050						
ECL_LB	10^{-4}	5308	1	0.02	0.02	265400						
	10^{-3}	39415	1	0.02	0.02	1970750						
	10^{-2}	389269	1	0.02	0.02	19463450						
LWECO_LB1e-4		3431	2	0.02	0.04	85775	337	8425	5875	0.05	13270	0.44
LWECO_LB1e-5		5887	2	0.02	0.04	147175	2305	57625	55075	0.05	98538	0.56
LWECO_LB1e-6		30870	2	0.02	0.04	771750	21781	544525	541975	0.05	973173	0.56
LWECO_MM1e-4		6080	2	0.02	0.04	152000	708	17700	15150	0.017	12538	1.21
LWECO_MM1e-5		8596	2	0.02	0.04	214900	3320	83000	80450	0.017	101024	0.80
LWECO_MM1e-6		43062	2	0.02	0.04	1076550	37772	944300	941750	0.017	731443	1.29

Section 4. Lake water microbial community enrichment

4.1 Analysis Fig. 3a and b

In order to evaluate the ANN classification of an unknown community, we incubated the Lake Geneva water microbial community with either phenol or 1-octanol, or without any further amendment, for three days. Microorganisms were collected from 10 L Lake Geneva water by filtration (0.2–40 µm pore size) taken in November 2018, and re-suspended in 100 ml ALW in acid-treated closed 500-ml glass Schott flasks to obtain starting cell concentrations of 10^5 cells ml^{-1}. Uniformly ^{14}C-labeled phenol or 1-C ^{14}C-labeled 1-octanol (ANAVA Trading SA) were dosed at 1000–5000 dpm ml^{-1} in a mixture with unlabeled compound of the same type, to obtain total carbon concentrations of 0.1, 1 or 10 mg C l^{-1}. Incubations with unlabeled phenol were further repeated three times independently with Lake Geneva microbial communities sampled in October and November 2017, and January 2019. Unamended inoculated ALW served as control for background growth, whereas amended but non-inoculated ALW served as abiotic controls. Triplicate flasks were prepared per assay, and incubated at 21 °C in the dark with 150 rpm rotary shaking. Aliquots of 1 ml were taken immediately after dosing the substrate, and then daily by syringes with needles without opening the caps, for cell staining with SYBR Green I and FCM analysis. FCM data were exported,

filtered and anchored as described above, and used as input for ANN classification using the five ANN-32 classifiers.

```
%open relevant files
%paths: /PHE_OCT_enrichments
%filter and log-transform as in section 1.1. Add anchors.
%classify with ANN-32 as in section 3.6
%take the mean and standard deviation. Plot in stack plot either as absolute counts per ml or
as relative counts normalized to the total number of counted cells per sample as in panel b.
```

4.2 Analysis of Fig. 3c

CellCognize part on the left. See above for procedure on the relevant comparison files.

16S rRNA gene amplicon sequenced diversity on same samples on the right.

Community diversity analysis by 16S rRNA gene amplicon sequencing. Lake Geneva water prokaryotic species diversity was determined by 16S rRNA gene amplicon sequencing. Samples from the enrichment experiment carried out with phenol and 1-octanol at 10 mg C l^{-1} in January 2019 as described above were collected immediately after dosing substrate and after three days incubation at room temperature in the dark. Sample volumes were adjusted to have similar cell densities at both time points. Cells were collected on 0.2-µm membrane filters (PES, Sartorius) and stored in FastDNA Spin kit solution for soil (MPBio) at -80 °C until analysis. Prior to DNA extraction, we added two internal standards (with cell number adjusted to 1% of the total cell density measured in the sample by FCM) to normalize 16S rRNA gene sequence variant abundances across samples, if necessary. The internal standards were in-house honeybee gut microbiota isolates belonging to *Giliamella* and *Bifidobacterium* (kindly provided by Lucie Kesnerova, University of Lausanne), which are unlikely to be found in lake water. After DNA extraction according to the manufacturer's recommendations for the FastDNA Spin kit for soil (MPBio), the V3–V4 hypervariable region of the 16S rRNA gene was amplified using the 341f/785r primer set with appropriate Illumina adapters and barcodes. PCR conditions, amplifications and library preparations followed recommendations in the Illumina Amplicon sequencing protocol (https://support.illumina.com/documents/documentation/chemistry_documentation/16s/16s-metagenomic-library-prep-guide-15044223-b.pdf). Equal amounts of amplified DNA from each sample were pooled and sequenced bidirectionally on the Illumina MiSeq platform at the University of Lausanne. Raw 16S rRNA gene amplicon sequences were separated by barcode, quality filtered, concatenated, verified for the absence of potential chimera, dereplicated and mapped to known bacterial classes (level 3) or species (level 7) using QIIME2 at 99% similarity to the SILVA taxonomic reference gene database on a UNIX platform[3].

4.3 Analysis of Fig. 3d

Alpha- and beta-diversity measures were calculated in *R* using the *phyloseq* package.

Compositional similarities between samples were calculated using Bray-Curtis dissimilarity index from relative abundance of each taxon and visualized in R by multidimensional scaling (MDS) analysis. Quantification of differences was performed using a Mantel test on the distance matrices from CellCognize and 16S rRNA gene amplicon, a Spearman correlation and a procrustes test of the MDS plots. Significance of replicate grouping was quantified using ADONIS.

Section 5. Analysis of similarity scores using CellCognize.

5.1 Calculate mean probabilities of standards in their class attribution

Calculate mean probabilities of the attribution of standards to their own class. Use a subsampled (n = 5000) dataset of all filtered and gated 32 standards. For each of the individual standards in that file, add the anchors, and classify using one of the classifier functions. Calculate the mean

probabilities of attribution to the correct class across all rows, but only if they are above 0.1. Plot the mean probabilities as orange bar plot in Fig. 4a top panel. As an example of the probability attributions per ‘event’ per class in the FCM file, see Datasheet 2. This is essentially summarized across all rows.

```
%% calculate mean probabilities of the attribution of standards to themselves.

%%

load('standard_normz_restricted_32.mat')

anchors=[2,2,2,2,2,2,1;6.6,6.6,5.7,6.3,6.3,6.0,3.3];

mean_probs=[]

for k=1:32
        input_community=vertcat(anchors,standard_normz{k});

        input_community=input_community';

        %when taking mean probabilities

        output_restricted_anchor1=
NNfunction_wide_anchor_normz_filtered_260619(input_community);
        max_probability=max(output_restricted_anchor1);
        output_restricted_anchor_final1=vec2ind(output_restricted_anchor1);

        %take mean across all rows individually, only if they are bigger than 0.1 (can change
this number) and place in a new column

        mean_h=zeros(32,1);
                for i=1:32
                h=output_restricted_anchor1(i,:);
                hl=h>0.1;
                he=h(hl);
                mean_he=mean(he);
                mean_h(i)=mean_he;
        end
        mean_probs{k}=mean_h;
end

m=[mean_probs{1} mean_probs{2} mean_probs{3} mean_probs{4} mean_probs{5} mean_probs{6}
mean_probs{7} mean_probs{8} mean_probs{9} mean_probs{10} mean_probs{11} mean_probs{12}
mean_probs{13} mean_probs{14} mean_probs{15} mean_probs{16} mean_probs{17} mean_probs{18}
mean_probs{19} mean_probs{20} mean_probs{21} mean_probs{22} mean_probs{23} mean_probs{24}
mean_probs{25} mean_probs{26} mean_probs{27} mean_probs{28} mean_probs{29} mean_probs{30}
mean_probs{31} mean_probs{32}];

m_red=[m(1,1);m(2,2);m(3,3);m(4,4);m(5,5);m(6,6);m(7,7);m(8,8);m(9,9);m(10,10);m(11,11);m(12,
12);m(13,13);m(14,14);m(15,15);m(16,16);m(17,17);m(18,18);m(19,19);m(20,20);m(21,21);m(22,22)
;m(23,23);m(24,24);m(25,25);m(26,26);m(27,27);m(28,28);m(29,29);m(30,30);m(31,31);m(32,32)];

%%save as excel
```

5.2 Calculate mean probability per assigned class in lakewater.

Grey values in Fig. 4a top panel. Lower part of Fig. 4a is the assigned class attribution from the lakewater samples using the ANN-32 classifier.

```
%change path

load('lakewaterlinear.mat')

%has 7 columns

%do the filtering and log transformation as in section 1.1

%regroup the columns back into one file

input_community=horzcat(scales1,scales2,scales3,scales4,scales5,scales6,scales7);

anchors=[2,2,2,2,2,2,1;6.6,6.6,5.7,6.3,6.3,6.0,3.3];
```

```
input_community=vertcat(anchors,input_community);

%transpose numeric array to be conform to NN input

input_community=input_community';

%run NN function
%NN example in /NN_file_example

output_restricted_anchor1= NNfunction_wide_anchor_normz_filtered_260619(input_community);
max_probability=max(output_restricted_anchor1);
output_restricted_anchor_final1=vec2ind(output_restricted_anchor1);

%%retain maximum per assigned cell

for i=1:5038;
        A=output_restricted_anchor1(:,i);
        C=max_probability(:,i);
        A(A<C)=0;
        output_restricted_anchor1(:,i)=A;
end


%%take means for all classes

for k=1:32
        mean_class(k)=mean(nonzeros(output_restricted_anchor1(k,:)));
        mean_class=mean_class';
end

%%save values of mean_class
```

5.3 Analysis of Fig. 4b. Probability distributions

In figure 4 b we plot the distribution of the assigned probability values per ‘event’ per selected class (for a data example, see Datasheet2). This is done here either for the community from lake water only, or from the lake water community data *in silico* mixed with data sets coming from the standards, or from the standards themselves. Reported means within panels are the simple mean of the retained values in the histograms.

```
%%%PART 2%%%
%%plot relevant attributed classes in the standard dataset above, for example
%%class 1=B02

A=output_restricted_anchor1(1,:);
B=A>0;
A=A(B);
        FigH=figure;
        height=100;
        width=100;
        x0=10;
        y0=10;
        p(1)=histogram(A,'Normalization','probability');
        p(1).BinWidth=0.05;
        set(gca,'fontsize',6)
        xlim([0,1]);
        ylim([0,1]);
        set(gcf,'position',[x0,y0,width,height]);
        xlabel('Probability', 'FontSize',6)
        ylabel('Density', 'FontSize',6)
        grid on
        filename=sprintf('GIVE_NAME%.4d.pdf',1);
        title(filename);
        saveas(FigH, filename,'pdf');


%%class 29=PVR

A=output_restricted_anchor1(29,:);
B=A>0;
A=A(B);
        FigH=figure;
        height=100;
        width=100;
```

```
        x0=10;
        y0=10;
        p(1)=histogram(A,'Normalization','probability');
        p(1).BinWidth=0.05;
        set(gca,'fontsize',6)
        xlim([0,1]);
        ylim([0,1]);
        set(gcf,'position',[x0,y0,width,height]);
        xlabel('Probability', 'FontSize',6)
        ylabel('Density', 'FontSize',6)
        grid on
        filename=sprintf('GIVE_NAME%.4d.pdf',29);
        title(filename);
        saveas(FigH, filename,'pdf');

%%CCR1 = 18
A=output_restricted_anchor1(18,:);
B=A>0;
A=A(B);
        FigH=figure;
        height=100;
        width=100;
        x0=10;
        y0=10;
        p(1)=histogram(A,'Normalization','probability');
        p(1).BinWidth=0.05;
        set(gca,'fontsize',6)
        xlim([0,1]);
        ylim([0,1]);
        set(gcf,'position',[x0,y0,width,height]);
        xlabel('Probability', 'FontSize',6)
        ylabel('Density', 'FontSize',6)
        grid on
        filename=sprintf('GIVE_NAME%4d.pdf',18);
        title(filename);
        saveas(FigH, filename,'pdf');


%%14=ACH2
A=output_restricted_anchor1(14,:);
B=A>0;
A=A(B);
        FigH=figure;
        height=100;
        width=100;
        x0=10;
        y0=10;
        p(1)=histogram(A,'Normalization','probability');
        p(1).BinWidth=0.05;
        set(gca,'fontsize',6)
        xlim([0,1]);
        ylim([0,1]);
        set(gcf,'position',[x0,y0,width,height]);
        xlabel('Probability', 'FontSize',6)
        ylabel('Density', 'FontSize',6)
        grid on
        filename=sprintf('GIVE_NAME%.4d.pdf',14);
        title(filename);
        saveas(FigH, filename,'pdf');

%% repeat for any class or sample, or in silico analyzed mixture, as desired. For in silico
mixing, follow, for example, section 3.7 or 3.9
```

5.4 Analysis of Fig. 4c. Mean class attribution and similarity scores in the 1-octanol enriched lake water community, using the ANN-32 classifiers.

Mean class attribution (absolute cell numbers) of the lake water enriched community on 1-octanol (n = 536,783 cells), and of the pure culture isolate (OCT, n = 63,824 cells) derived from this enrichment grown on 1-octanol, both after three days of incubation, for one of the ANN-32 classifiers and for a new classifier that was trained 759 using a dataset that in addition included FCM data from the OCT isolate itself (ANN-33). Numbers on the bars indicate the mean probability of class attribution.

Panels on the left part, analysis using the ANN-32 classifier.

```
% go to PHE_OCT_enrichments/PHE3_OCT1/T3/

LWECO1=readtable('Specimen1_A6_10 C Octanol 1.csv');
LWECO1=table2array(LWECO1);
LWECO2=readtable('Specimen1_B6_10 C Octanol 2.csv');
LWECO2=table2array(LWECO2);
LWECO3=readtable('Specimen1_C6_10 C Octanol 3.csv');
LWECO3=table2array(LWECO3);

community_combined=vertcat(LWECO1,LWECO2,LWECO3);

%this has the concatenated OCT files from FCM, but not filtered nor log-transformed

%remove 8th columns

community_combined(:,8)=[];


%do the filtering and log transformation as in section 1.1

%regroup the columns back into one file

input_community=horzcat(scales1,scales2,scales3,scales4,scales5,scales6,scales7);

%add anchors

anchors=[2,2,2,2,2,2,1;6.6,6.6,5.7,6.3,6.3,6.0,3.3];


input_community=vertcat(anchors,input_community);
input_community=input_community';

%%classify with the ANN32-standard classifier

%NN example in /NN_file_example

output_restricted_anchor1= NNfunction_wide_anchor_normz_filtered_260619(input_community);
max_probability=max(output_restricted_anchor1);
output_restricted_anchor_final1=vec2ind(output_restricted_anchor1);

%%calculate class attribution

classes=(0:33);
empty_class=[33;0];

membership_restricted1 =
histc(output_restricted_anchor_final1,unique(output_restricted_anchor_final1));

classes_restricted1=unique(output_restricted_anchor_final1);
final_community1= [classes_restricted1;membership_restricted1];

finalComm1=[final_community1,empty_class];

%apply a logical function to find corresponding values in the list with all categories in the
file 'classes'

[lic,loc]=ismember(classes,finalComm1(1,:));
results1(2,lic)=finalComm1(2,loc(lic));
results1(1,:)=classes;

results=results1';

%% save results (is class attribution)

%%retain maximum probability per assigned cell/’event’

for i=1:536783;
                A=output_restricted_anchor1(:,i);
                C=max_probability(:,i);
                A(A<C)=0;
                output_restricted_anchor1(:,i)=A;
end

%%%%take means for all classes

for k=1:32
```

```
        mean_class(k)=mean(nonzeros(output_restricted_anchor1(k,:)));
        mean_class=mean_class';
end

% save values of mean_class
```

Repeat the analysis, but now for the FCM data of the OCT isolate

```
% go to PHE_OCT_enrichments/OCT_PHE_isolates/T3/


LWECO1=readtable('Specimen7_B1_OCT2,0.csv');
LWECO1=table2array(LWECO1);
LWECO2=readtable('Specimen7_C1_OCT3,0.csv');
LWECO2=table2array(LWECO2);
LWECO3=readtable('Specimen7_D1_OCT4,0.csv');
LWECO3=table2array(LWECO3);

community_combined=vertcat(LWECO1,LWECO2,LWECO3);

%Then continue as for the above in section 1.1 and 5.4
```

5.5 Mean class attribution and similarity scores using an ANN-33 classifier

Analysis for the right part of the figure 4c. First produce a classifier that includes the OCT-isolate itself

```
load('filtered_standards_32.mat')

%prepare OCT4 standard

OCT4=readtable('Specimen7_D1_OCT4,0.csv');
OCT3=readtable('Specimen7_C1_OCT3,0.csv');
OCT2=readtable('Specimen7_B1_OCT2,0.csv');
community_combined=vertcat(OCT4,OCT3,OCT2);

%remove 8th column

community_combined(:,8)=[];

community_combined=table2array(community_combined);

%filtering and log transformation as in section 1.1

%regroup the columns back into one file

input_community=horzcat(scales1,scales2,scales3,scales4,scales5,scales6,scales7);

%rename to OCT

OCT=input_community;

%add OCT to filtered standards, making a 33 standard set.

filtered_standards={B02,B05,B1,B10,B15,B2,B4,B6,AJH1,AJH2,ATJ1,ATJ2,ACH1,ACH2,ACH3,BST1,BST2,
CCR1,CCR2,CAL,ECL_EXP3,ECL_STAT_LB,ECL_STAT_MM,ECL,LLC,PKM1,PMG,PPT,PVR1,PVR2,SWT,SYN,OCT};

%then continue as in section 2.1 and 2.2 to traing, validate and test the ANN classifier.
```

Repeat the analysis from above of the OCT enrichment and the OCT isolate using the new ANN33 classifier. Fig. 4c right part.

```
%%continue with the same data sets as above in section 5.4

%%classify using ANN33-standard classifier, produced in section 5.5, first part above.
Example NN file in /NN_file_example


output_restricted_anchor1= NNfunction_wide_anchor_normz_filtered_OCT_170819(input_community);
max_probability=max(output_restricted_anchor1);
output_restricted_anchor_final1=vec2ind(output_restricted_anchor1);

%%recalculate class attribution
```

```
classes=(0:34);
empty_class=[34;0];

membership_restricted1 =
histc(output_restricted_anchor_final1,unique(output_restricted_anchor_final1));

classes_restricted1=unique(output_restricted_anchor_final1);
final_community1= [classes_restricted1;membership_restricted1];

finalComm1=[final_community1,empty_class];

%apply a logical function to find corresponding values in the list with all categories in the
file 'classes'

[lic,loc]=ismember(classes,finalComm1(1,:));
results1(2,lic)=finalComm1(2,loc(lic));
results1(1,:)=classes;

results=results1';

%% save values of results

%%retain maximum probability per assigned cell

for i=1:536783;
               A=output_restricted_anchor1(:,i);
               C=max_probability(:,i);
               A(A<C)=0;
               output_restricted_anchor1(:,i)=A;
end

%%%%take means for all classes

for k=1:33
mean_class(k)=mean(nonzeros(output_restricted_anchor1(k,:)));
mean_class=mean_class';
end

%%%%% save values of mean_class


%plot probability histogram of relevant class 33 OCT

A=output_restricted_anchor1(33,:);
B=A>0;
A=A(B);
       FigH=figure;
       height=100;
       width=100;
       x0=10;
       y0=10;
       p(1)=histogram(A,'Normalization','probability');
       p(1).BinWidth=0.05;
       set(gca,'fontsize',6)
       xlim([0,1]);
       ylim([0,1]);
       set(gcf,'position',[x0,y0,width,height]);
       xlabel('Probability', 'FontSize',6)
       ylabel('Density', 'FontSize',6)
       grid on
       filename=sprintf('GIVE_NAME%.4d.pdf',33);
       title(filename);
       saveas(FigH, filename,'pdf');

% repeat the above for the OCT isolate data itself.
```

Supplementary Reference

1. https://ch.mathworks.com/help/deeplearning/ref/plotconfusion.html.

2. Loferer-Krossbacher, M., Klima, J. & Psenner, R. Determination of bacterial cell dry mass by transmission electron microscopy and densitometric image analysis. *Appl Environ Microbiol* **64**, 688-694 (1998).
3. Bolyen, E. *et al.* Reproducible, interactive, scalable and extensible microbiome data science using QIIME 2. *Nat Biotechnol* **37**, 852-857 (2019).
