# Supplementary material for: Rapid detection of microbiota cell type diversity using machine-learned classification of flow cytometry data
Source: Commun Biol. 2020 Jul 15;3:379. doi: 10.1038/s42003-020-1106-y (PMC7363847; doi:10.1038/s42003-020-1106-y)
Supplement: Supplementary file 20 — Description of Additional Supplementary Files [file 42003_2020_1106_MOESM20_ESM.pdf]

Supplementary Data 1: shows the average recall and precision of the neural network training, from five independently generated classifiers

Supplementary Data 2: shows an example of the probability scoring for particles in unknown

communities, for the first 20 particles in the dataset.

Supplementary Data 3: Source data for Figure 2 panel a

Supplementary Data 4: Source data for Figure 2 panel b

Supplementary Data 5: Source data for Figure 2 panel d

Supplementary Data 6: Source data for Figure 2 panel d

Supplementary Data 7: Source data for Figure 2 panel e

Supplementary Data 8: Source data for Figure 2 panel f

Supplementary Data 9: Source data for Figure 3 panel a

Supplementary Data 10: Source data for Figure 3 panel c, 16S taxonomies

Supplementary Data 11: Source data for Figure 3 panel c, ANN taxonomies

Supplementary Data 12: Source data for Figure 3 panel d: Shannon and Richness

Supplementary Data 13: Source data for Figure 3 panel d: NMDS

Supplementary Data 14: Source data for Figure 4 panel a

Supplementary Data 15: Source data for Figure 4 panel b

Supplementary Data 16: Source data for Figure 4 panel c

Supplementary Data 17: Source data for Table 2.
